# Fasting-mimicking diet plus chemotherapy in breast cancer treatment

**DOI:** 10.1038/s41467-020-18194-1

**Published:** 2020-08-26

**Authors:** Claudio Vernieri, Francesca Ligorio, Emma Zattarin, Licia Rivoltini, Filippo de Braud

**Affiliations:** 1 Medical Oncology and Hematology Department, Fondazione IRCCS Istituto Nazionale dei Tumori, Via Venezian 1, 20133 Milan, Italy; 2 Metabolic Reprogramming in Solid Tumors Unit. IFOM, the FIRC Institute of Molecular Oncology, Via Adamello 16, 20139 Milan, Italy; 3 Unit of Immunotherapy of Human Tumors, Fondazione IRCCS Istituto Nazionale dei Tumori, Via Venezian 1, 20133 Milan, Italy; 4 Oncology and Hemato-Oncology Department, University of Milan, 20122 Milan, Italy

**Keywords:** Breast cancer, Cancer metabolism

## Abstract

A clinical trial published in *Nature Communications* examined the effect of fasting-mimicking diet (FMD) during chemotherapy in breast cancer patients. The overall negative study results highlight the need for ameliorating future trial design and investigating alternative FMD-based therapeutic combinations.

## The “DIRECT” trial

In a study recently published in *Nature Communications*, de Groot et al. reported the results of the phase II trial “DIRECT”, in which experimental cyclic fasting-mimicking diet (FMD) in combination with standard anthracycline-taxane preoperative chemotherapy (ChT) failed to reduce ChT-related adverse events and to improve the rate of pathological complete responses (pCR) in patients with stage II–III HER2-negative breast cancer (BC)^1^. However, some study findings, such as the possibility to avoid dexamethasone use during doxorubicin-cyclophosphamide ChT and the reduction of ChT-induced DNA damage to circulating lymphocytes in patients undergoing the FMD, are of potential interest. While highlighting the importance of publishing results of clinical trials even when they are negative for their primary endpoint, findings of the DIRECT trial indicate the necessity to ameliorate patient adherence to the FMD and to improve clinical trial designs to fully exploit the therapeutic potentialities of calorie-restricted dietary interventions.

In tumor-bearing mice, cycles of fasting or calorie-restricted, low-carbohydrate, low-protein diets, collectively referred to as FMDs, synergize with cytotoxic ChT or other antitumor therapies to slow down tumor growth^2,3^. At the same time, fasting/FMD protect normal tissues from ChT-induced toxicity^4,5^ and boosts ChT-induced CD8+ T cell intratumor infiltration^2^. These effects are mainly mediated by fasting/FMD-induced reduction of blood glucose, insulin and insulin-like growth factor 1 (IGF-1) concentration^4,5^. Notably, murine models of BC are exquisitely sensitive to the FMD when compared to other tumor types^2,3^. Therefore, the DIRECT trial, which investigated the FMD efficacy in reducing ChT-induced toxicities and in increasing pCR rates in HER2- BC patients, was timely and based on solid preclinical evidence. In addition, the study was conducted in early-stage BC patients, who are at low risk of undergoing malnutrition and cachexia,. Finally, this was a randomized phase II/III trial with sufficient power to provide evidence of a clinical benefit of the FMD in a selected population of cancer patients. For these reasons, the DIRECT trial had the potential to provide proof-of-concept demonstration that the FMD positively impacts on ChT tolerability and antitumor activity in BC patients.

Unfortunately, the study was prematurely interrupted because a pre-planned interim analysis showed a lower-than-anticipated pCR rate in the study population, as well as poor patient adherence to the proposed FMD regimen^1^. In addition, the FMD did not provide evident clinical advantages, neither in terms of reduction of AEs (the primary endpoint of the phase II trial) nor in terms of increased pCR rates (the primary endpoint of the phase III trial)^1^. Of note, triple-negative breast cancer (TNBC) patients, who are much more likely to undergo pCR during preoperative ChT^6^, were significantly more represented in the FMD arm than in the control arm (21.5% vs. 10.9%). Therefore, an increase of pCR rates in the experimental arm would have been expected also independently of the FMD. Although the authors emphasized the occurrence of higher rates of clinical/radiological tumor responses in patients receiving the ChT-FMD combination, the clinical relevance of this finding is questionable. Indeed, while pCR is a suitable surrogate biomarker of preoperative ChT efficacy and a predictor of long-term clinical outcomes^6^, radiological responses are only poorly-to-moderately associated with pCR^7^, and their impact on long-term outcomes is uncertain.

## Improving patient adherence to the FMD: a crucial issue

One reason that could explain the negative results of the DIRECT trial is low patient compliance with the experimental FMD regimen. Indeed, although the maximum number of allowed FMD cycles was 8 as per protocol, only ~50% and 33.8% of patients were able to complete at least 2 and 4 FMD cycles, respectively. Such low compliance rates might have crucially contributed to the failure to reduce ChT-induced adverse events and to increase pCR rates in the experimental arm.

In mouse models, two fasting/FMD cycles in combination with ChT can be sufficient to slow-down tumor growth in the short-term period^2^, but a higher number of FMD cycles is required to achieve long-term tumor control. In the case of human cancers, which are characterized by higher biological complexity and heterogeneity when compared to smaller murine neoplasms^8^, completing a higher number of FMD cycles might be even more important to observe some clinical benefit. Consistent with the concept that patient adherence to the FMD is a crucial prerequisite for its antitumor activity, a per-protocol sub-analysis of the DIRECT trial showed that compliant patients had significantly higher rates of pathological tumor responses according to the Miller-Payne 4/5 scores (which may approximate pCR as a predictor of tumor recurrences limited to TNBC subgroup^9^), and also reported lower DNA damage to peripheral blood lymphocytes.

The main cause of lack of adherence to the experimental diet was dislike of specific components of the FMD, which consisted of a plant-based, standardized kit providing ~1200 kcal on day 1 and ~200 kcal on days 2–4^1^. Alternative FMD regimens, including schemes containing fresh foods, could be associated with better patient compliance, thus potentially resulting in higher antitumor activity^10^. Four clinical trials are currently being conducted at our institution to investigate the safety, feasibility, metabolic, immunological, and antitumor activity of cyclic FMD in combination with standard therapies in patients with different tumor types (ClinicalTrials.gov Identifiers: NCT03340935; NCT03454282; NCT03709147; NCT04248998). The FMD scheme used in these studies, which provides ~600 kcal on day 1 and ~300 kcal on days 2–5, consists of a list of permitted fresh foods and beverages, which patients can choose according to their preferences. Results of ongoing studies (Table 1) will clarify if more flexible and physiological FMD schemes can guarantee better patient compliance, thus becoming the reference FMD regimen to be tested in future clinical trials.

**Table 1:** Ongoing clinical trials employing FMDs in cancer patients.

Clinicaltrials.gov registration number	Title of the registered study	Status	Study design	Primary endpoint	Clinical setting	*n*	Type and schedule of FMD regimen	Study sponsor
NCT04292041	Fasting Mimicking Diet in Prostate Cancer and Metabolic Syndrome, a Pilot Study	Active, not recruiting	Prospective, single-arm trial	Change in baseline weight, blood pressure, waist circumference, triglycerides, Total, LDL, and HDL Cholesterol at 6 months	Prostate cancer patients with metabolic syndrome receiving standard treatment options	40	Chemolieve® for 3 cycles	Galway Clinic, Galway, Ireland
NCT03340935	Safety, Feasibility and Metabolic Effects of the Fasting Mimicking Diet (FMD) in Cancer Patients	Recruiting	Monocentric, single-arm, phase I/II trial	Safety of FMD, as defined as incidence of severe, FMD-related adverse events	Malignant neoplasms	95	5-day plant-based, low-calorie (600 Kcal on day 1, followed by 300 KCal/day on days 2–5), low-protein, low-carbohydrate diet composed of fresh food concomitantly with standard antitumor therapies	Fondazione IRCCS Istituto Nazionale dei Tumori, Milan, Italy
NCT03595540	Phase II Clinical Study of a Fasting-Mimicking Diet in Patients Undergoing Oncologic Treatment	Recruiting	Monocentric, single arm, phase I/II trial	Feasibility and safety of FMD	Solid or hematologic tumors undergoing active treatment	60	Prolon by L-Nutra concomitantly with standard antitumor therapies	University of Genova, Italy
NCT03700437	Randomized Controlled Pilot Study to Evaluate Fasting-mimicking Diet in Patients Receiving Chemo-immunotherapy for Treatment of Metastatic Non-small Cell Lung Cancer	Recruiting	Open Label, Randomized, phase II controlled trial	-Changes in CTCs -DNA damage in CTCs -PBMC profiles	Stage IV Lung Adenocarcinoma for which combined chemo- immunotherapy in the form of carboplatin/pemetrexed and pembrolizumab is being utilized.	40	Chemolieve® for 3 days prior to and on the day of chemo-immunotherapy during the first 4 cycles	Indiana University, USA
NCT03709147	Exploiting Metformin Plus/Minus Cyclic FAsting Mimicking Diet (FMD) to Improve the Efficacy of Platinum-pemetrexed Chemotherapy in Advanced LKB1-inactive Lung Adenocarcinoma: the FAME Trial	Not yet recruiting	Single Institution, open-label, double arm, non-comparative, randomized, single-stage phase II trial, with “pick-the-winner” design	Progression-free survival	Stage IV LKB1- inactive Lung Adenocarcinoma	88	5-day plant-based, low-calorie (600 Kcal on day 1, followed by 300 KCal/day on days 2–5), low-protein, low-carbohydrate diet composed of fresh food concomitantly with standard antitumor therapies, to be repeated every 3 weeks up to a maximum of 4 cycles	Fondazione IRCCS Istituto Nazionale dei Tumori, Milan, Italy
NCT03454282	Impact of Dietary Intervention on Tumor Immunity: the DigesT Trial	Recruiting	Monocentric, single-arm with three cohorts of patients	Absolute and relative changes in PBMCs	-Invasive breast cancer candidate to upfront curative surgery (Cohort A) -resected stage III melanoma (Cohort B) -resected, stage IIB/IIC melanoma (Cohort C)	100	5-day plant-based, low-calorie (600 Kcal on day 1, followed by 300 KCal/day on days 2–5), low-protein, low-carbohydrate diet composed of fresh food concomitantly with standard antitumor therapies	Fondazione IRCCS Istituto Nazionale dei Tumori, Milan, Italy
NCT04248998	Targeting Triple Negative BREAst Cancer Metabolism With a Combination of Chemotherapy and a Diet Mimicking FASTingPlus/Minus Metformin in the Preoperative Setting: the BREAKFAST Trial	Recruiting	Single center, open-label, double arm, randomized, single stage, phase II trial	pCR rate	Triple Negative Breast Cancer candidate to neoadjuvant chemotherapy	90	5-day plant-based, low-calorie (600 Kcal on day 1, followed by 300 KCal/day on days 2–5), low-protein, low-carbohydrate diet composed of fresh food concomitantly with standard antitumor therapies	Fondazione IRCCS Istituto Nazionale dei Tumori, Milan, Italy
NCT04027478	Can Fasting Decrease the Side Effects of Chemotherapy?	Enrolling by invitation	Open label, prospective randomized crossover trial.	Incidence of grade II/III/IV nausea	Patients undergoing chemotherapy with Taxol/carboplatin planned for at least 6 cycles	39	FMD consisting of 10 Kcal/kg/day and including 50% fat, 40% carbohydrates, and no more than 10% protein. The diet includes nuts, olives, vegetable broth, broccoli/cauliflower, white rice/puffed rice cake, onion, tea/coffee, almond milk. The diet prohibits meat products, dairy, alcohol, sugar, and artificial sweeteners.	Sutter Cancer Center, Sacramento, California, United States
NCT02710721	Clinical Study on the Efficacy of Fasting and Nutritional Therapy as a Complementary Treatment of Advanced Metastatic Prostate Cancer Undergoing Chemotherapy—an Exploratory Randomized Controlled Trial	Recruiting	Open Label, Randomized trial	FACT-P/-Taxane/-An sum score	Castration-resistant prostate cancer or hormone-sensitive metastatic prostate cancer with high disease burden	60	A 60 h-modified fasting (36 h before and 24 h after chemotherapy) with a dietary energy supply 350–400 kcal per day with fruit and vegetable juices or, if not feasible, an established FMD of 600-800 kcal (Chemolieve®)	Charité Centrum Chirurgische Medizin, CC 8 Klinik für Urologie, Berlin, Germany

Chemolieve® is a 4-day plant based low amino-acid substitution dietary kit, consisting of soups, broths, liquids, and tea

Prolon by L-Nutra is a 5-day dietary kit. Day 1 of Prolon provides ~4600 kJ (11% protein, 46% fat, and 43% carbohydrate), whereas days 2-to-5 provide ~3000 kJ (9% protein, 44% fat, and 47% carbohydrate) per day.

CTC, circulating tumor cells; FACT-P, functional assessment of cancer therapy-prostate cancer; FMD, fasting-mimicking diet; pCR, pathologic complete response; PBMC, peripheral blood mononuclear cells.

In parallel with poor compliance with experimental dietary regimens, another limitation of clinical trials investigating dietary interventions consists in the fact that patients randomized to the control diet can spontaneously reduce their calorie intake, thus confounding the study findings. In the DIRECT trial, only 7.8% of patients in the control arm admitted that they decided to fast, thus making it unlikely that their deviations impacted on the study findings. However, due to the relevance of this issue, alternative study designs, including cross-over trials, or randomized trials employing placebo diets with similar appearance to FMD kits, but with calorie and macronutrient content that is consistent with dietary recommendations for cancer patients (https://www.wcrf.org/dietandcancer/recommendations-about)^11^, should be considered in future trials.

## FMD to spare dexamethasone use and boost antitumor immunity

One interesting finding of the DIRECT trial is that patients undergoing the FMD did not receive dexamethasone as a premedication to doxorubicin-cyclophosphamide (AC) ChT, and yet they did not experience a higher incidence of AEs when compared to patients in the control arm. These data indirectly suggest that the FMD could protect patients from some ChT-induced AEs (e.g. nausea, vomiting), thus making dexamethasone premedication unnecessary. Since dexamethasone could promote BC metastasis^12^ and result in potentially detrimental metabolic and immunological effects^13^, avoiding dexamethasone administration could per se enhance ChT-induced anticancer effects in specific clinical contexts. Although intriguing, this hypothesis needs to be tested in future prospective trials.

Preclinical in vivo studies showed that two fasting/FMD cycles boost antitumor immunity and promote intratumor infiltration by CD8^+^ T cells through reducing blood IGF-1 levels, which is an essential determinant of the anticancer effects of calorie restriction^2^. If this also applies to human cancers, 1–2 FMD cycles, which were completed by the majority of patients in the DIRECT trial, might be sufficient to activate antitumor immunity. In this perspective, the observed reduction of DNA damage to circulating lymphocytes in compliant patients is interesting. If the FMD boosts antitumor immunity in cancer patients similarly to tumor-bearing mice, FMD-induced clinical benefit might become apparent in the long-term period, thus resulting in reduced tumor recurrences and improved long-term outcomes despite the lack of pCR advantages. In this perspective, it will be interesting to analyze relapse-free survival and overall survival (OS) data from the DIRECT study when they are mature.

## How to improve FMD antitumor efficacy in future trials?

FMD antitumor activity could be attenuated by adaptive metabolic mechanisms allowing cancer cells to survive also in conditions of glucose and growth factor deprivation. For instance, cancer cells can adapt to fasting/FMD-induced reduction of extracellular glucose by increasing the utilization of mitochondrial oxidative phosphorylation (OXPHOS) as an alternative source of energy units (ATP) and anabolic precursors. One recent study showed that the simultaneous inhibition of tumor glycolysis (through cycling fasting) and OXPHOS (through metformin) produces synergistic and long-lasting anticancer effects in tumor cell lines and tumor-bearing mice, which are mediated through a potent activation of the PP2A-GSK3β tumor suppressor axis^14^. Based on these data, we have recently initiated the phase II, randomized study “BREAKFAST” (NCT04248998) to test the anticancer activity of adding cyclic FMD, or FMD plus metformin, to standard preoperative ChT in stage I-III TNBC patients (Table 1).
